# Leveraging Electronic Health Records in International Humanitarian Clinics for Population Health Research: Cross-Sectional Study

**DOI:** 10.2196/66223

**Published:** 2025-04-17

**Authors:** Sarah Draugelis, Jessica Hunnewell, Sam Bishop, Reena Goswami, Sean G Smith, Philip Sutherland, Justin Hickman, Donald A Donahue, George A Yendewa, Amir M Mohareb

**Affiliations:** 1 Team fEMR, St. Clair Shores, MI, United States; 2 Center for Global Health, Massachusetts General Hospital, 125 Nashua St, #722, Boston, MA, 02114, United States; 3 University of Pittsburgh School of Medicine, Pittsburgh, PA, United States; 4 Global Response Medicine, Marco Island, FL, United States; 5 Department of Medicine, Massachusetts General Hospital, Boston, MA, United States; 6 Critical-Care Professionals International, Graham, FL, United States; 7 World Association for Disaster and Emergency Medicine, Madison, WI, United States; 8 Beth Israel Deaconess Medical Center, Boston, MA, United States; 9 Faculty of Medicine, Sigmund Freud Private University, Vienna, Austria; 10 Department of Medicine, Case Western Reserve University, Cleveland, OH, United States; 11 Division of Infectious Diseases and HIV Medicine, University Hospitals Cleveland Medical Center, Cleveland, OH, United States; 12 Department of Medicine, Harvard Medical School, Boston, MA, United States

**Keywords:** refugee, population health, disaster medicine, humanitarian clinic, electronic health record, Fast Electronic Medical Record, fEMR

## Abstract

**Background:**

As more humanitarian relief organizations are beginning to use electronic medical records in their operations, data from clinical encounters can be leveraged for public health planning. Currently, medical data from humanitarian medical workers are infrequently available in a format that can be analyzed, interpreted, and used for public health.

**Objectives:**

This study aims to develop and test a methodology by which diagnosis and procedure codes can be derived from free-text medical encounters by medical relief practitioners for the purposes of data analysis.

**Methods:**

We conducted a cross-sectional study of clinical encounters from humanitarian clinics for displaced persons in Mexico between August 3, 2021, and December 5, 2022. We developed and tested a method by which free-text encounters were reviewed by medical billing coders and assigned codes from the *International Classification of Diseases, Tenth Revision* (*ICD-10*) and the *Current Procedural Terminology* (*CPT*). Each encounter was independently reviewed in duplicate and assigned *ICD-10* and *CPT* codes in a blinded manner. Encounters with discordant codes were reviewed and arbitrated by a more experienced medical coder, whose decision was used to determine the final *ICD-10* and *CPT* codes. We used chi-square tests of independence to compare the *ICD-10* codes for concordance across single-diagnosis and multidiagnosis encounters and across patient characteristics, such as age, sex, and country of origin.

**Results:**

We analyzed 8460 encounters representing 5623 unique patients and 2774 unique diagnosis codes. These free-text encounters had a mean of 20.5 words per encounter in the clinical documentation. There were 58.78% (4973/8460) encounters where both coders assigned 1 diagnosis code, 18.56% (1570/8460) encounters where both coders assigned multiple diagnosis codes, and 22.66% (1917/8460) encounters with a mixed number of codes assigned. Of the 4973 encounters with a single code, only 11.82% (n=588) had a unique diagnosis assigned by the arbitrator that was not assigned by either of the initial 2 coders. Of the 1570 encounters with multiple diagnosis codes, only 3.38% (n=53) had unique diagnosis codes assigned by the arbitrator that were not initially assigned by either coder. The frequency of complete concordance across diagnosis codes was similar across sex categories and ranged from 30.43% to 46.05% across age groups and countries of origin.

**Conclusions:**

Free-text electronic medical records from humanitarian relief clinics can be used to develop a database of diagnosis and procedure codes. The method developed in this study, which used multiple independent reviews of clinical encounters, appears to reliably assign diagnosis codes across a diverse patient population in a resource-limited setting.

## Introduction

The global population of forcibly displaced persons, including refugees, asylum seekers, and internally displaced persons, has surpassed 100 million people [1]. Both human-made and natural disasters can break down traditional centers of health care delivery, which may leave displaced persons with gaps in medical care [2]. Displaced persons who leave their countries of origin are often highly mobile between the time of displacement and resettlement [1], which can create challenges for continuity of care, including management of chronic medical conditions, provision of psychiatric and mental health services, and prevention and treatment of communicable diseases.

Humanitarian organizations that provide medical care for displaced persons do so with few resources, limited technological infrastructure, and high patient and staff turnover [3]. Staff turnover may pose serious challenges to maintaining continuity of care for displaced persons. Health information, if properly leveraged, can guide public health interventions for these populations [4,5]. More humanitarian relief organizations are transitioning their operations from paper to electronic medical records, but clinical documentation in ad hoc relief clinics is typically not standardized, nor conducive to analysis [2]. Specifically, clinicians working in resource-limited settings, particularly in disaster response scenarios, may not have time to consistently document all aspects of history-taking and the physical examination, and they may not themselves formally assign *International Classification of Diseases, Tenth Revision* (*ICD-10*) codes to patient encounters [4,5]. Quality of care in these settings can be greatly enhanced if data from clinical documentation can be effectively analyzed and interpreted [6,7]. Electronic health records designed for austere environments may also be an untapped resource to enhance population health and guide public health interventions, for example, by contributing to systems of syndromic surveillance for early warning of infectious disease outbreaks in humanitarian settings [7-9].

Past research has evaluated the ability for free-text clinical documentation to be analyzed with high efficiency, both by manual review and using natural language processing [10,11]. Studies using machine learning methods have used free-text clinical documentation, mostly from high-income settings, to categorize symptom presentations and classify diagnosed chronic diseases [11,12]. However, few studies have evaluated free-text clinical documentation in humanitarian relief settings where clinicians are under particularly high time pressures and operate with limited resources. There is a growing recognition that clinical documentation from humanitarian relief settings could provide much-needed data to measure the outcomes and improve the performance of humanitarian action [13]. To address this gap, we developed and tested a methodology of assigning medical diagnosis codes to free-text medical records collected from a humanitarian clinic at the Mexico-US border.

## Methods

### Study Design

We conducted a cross-sectional study of clinical encounters from humanitarian clinics for displaced persons at the Mexico-US border between August 3, 2021, and December 5, 2022. There has been a rise over the past decade in the number of displaced persons who transit through the Rio Grande Valley, which is situated near the Eastern coast of the Mexico-US border. Volunteer clinicians with Global Response Medicine, an international humanitarian relief organization, documented medical encounters using Fast Electronic Medical Record (fEMR), a digital medical record system specifically designed for disaster response [14]. Details of a similar clinical operation are separately described [15]. We created a program in which trained volunteer medical coders reviewed free-text medical records from this clinical site and assigned *ICD-10* and *Current Procedural Terminology* (*CPT*) codes. Our analysis included all the encounters with completed medical triage in the camp (ie, the patient registered in the electronic system) and which were assigned a medical diagnosis by the coders.

### Ethical Considerations

All fEMR records used in this study were deidentified and made Health Insurance Portability and Accountability Act–compliant to protect patient privacy. Informed consent and participant compensation for research were not possible as all data were deidentified. No research participant was recruited for this study as it was a review of medical records. We did not use machine learning, artificial intelligence, language models, or any similar technology for the purposes of information gathering, analyses, content creation, manuscript writing, or manuscript editing. This study was approved by the fEMR board of directors and the Mass General Brigham Institutional Review Board (2022P003273).

### Training of Medical Coders

Coders underwent a rigorous onboarding process before participating in the project. As a prerequisite, they were required to achieve a score of 70% accuracy or higher on a structured entry examination, as determined by experienced medical coders who designed the training. This examination used a subset of medical records from the study dataset to assess the coder’s ability to accurately interpret and assign codes in alignment with the project’s standards. In addition, all coders were required to complete training in Health Insurance Portability and Accountability Act to ensure compliance with patient privacy and data security regulations.

To support coders in their tasks and ensure uniformity in the coding process, they were provided with access to a comprehensive set of resources. These resources included detailed instructions on the coding process, examples of coded records, and a manual specifically tailored to the unique aspects of the dataset used in this study. This preparation was designed to standardize the approach across coders, reducing variability and enhancing the reliability of the coding process.

### Assignment of Diagnosis Codes

To ensure consistency and reliability in the assignment of *ICD-10* and *CPT* codes, a structured process was developed for the assignment of coders. All coders were randomly assigned blocks of 100 clinical records to review and code. Each block was to be completed within 30 days of assignment. Once a coder submitted their completed block, they were assigned the next available block of 100 records in the dataset. This approach minimized the potential for systematic biases related to the order of record review, such as rater fatigue. Importantly, there were no penalties for exceeding the 30-day completion period, nor incentives for finishing ahead of schedule, ensuring that coders could focus on accuracy rather than speed.

**Figure 1.**
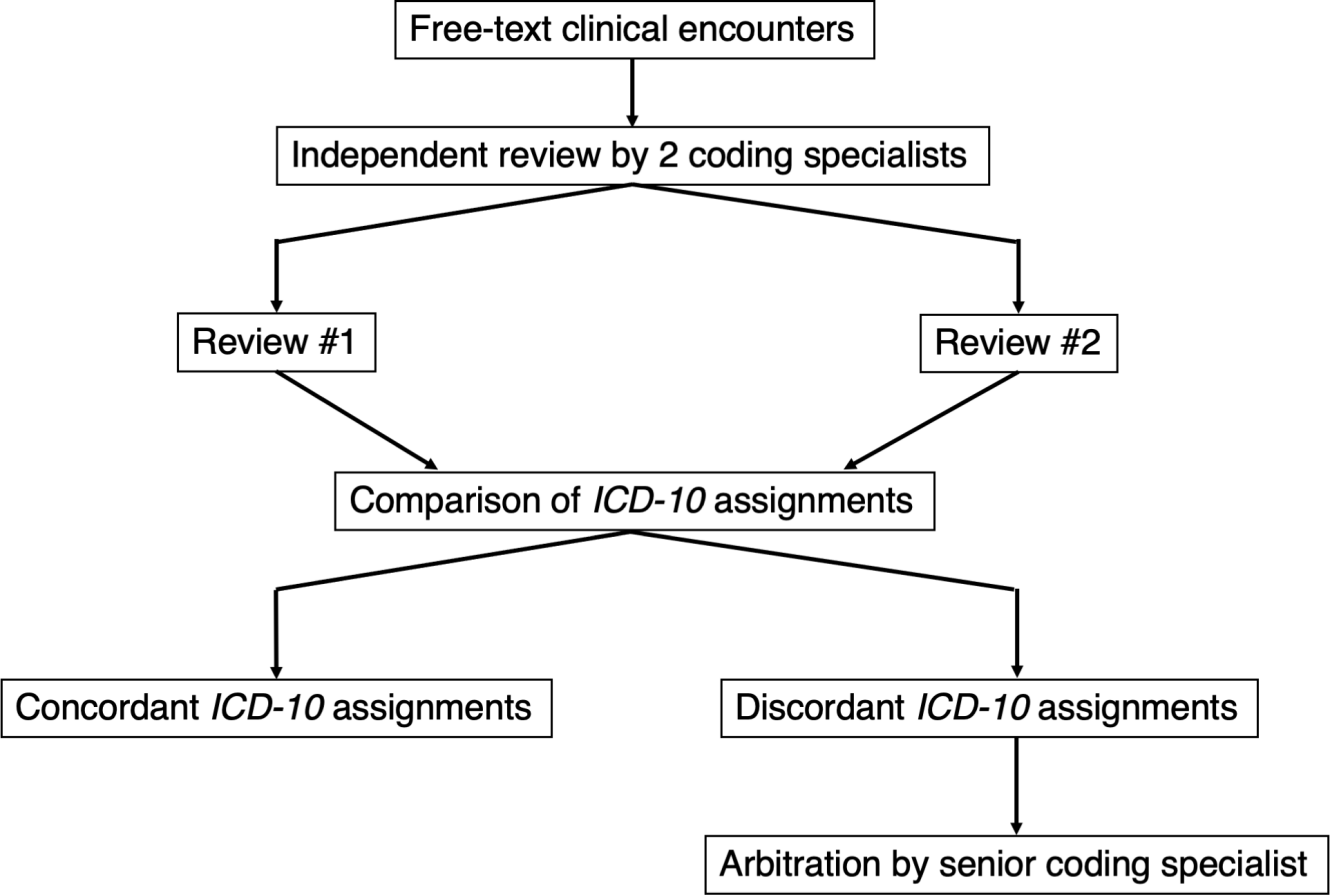
Workflow for assignment of *ICD-10* codes to free-text clinical encounters from humanitarian relief clinics in Mexico, 2021-2022. *ICD-10*: *International Classification of Diseases, Tenth Revision*.

Each record was independently reviewed by 2 coders who assigned *ICD-10* and *CPT* codes in a manner blinded to each other. We compared the codes for concordance, which we defined as an exact match in both the *ICD-10* codes. When discrepancies were identified, the arbitration process was initiated. Specifically, a third, more experienced coder, who had undergone additional training in advanced medical coding, reviewed the original free-text record along with the codes assigned by both initial coders. This coder then independently determined the final coding assignment based on established guidelines and standards for *ICD-10* and *CPT* coding. The final arbitration was documented along with necessary notes detailing the rationale for code selection to facilitate quality assurance and reproducibility in future applications of the process (Figure 1). Quarterly team calls were conducted to review coding processes and provide clarification on complex cases. In addition, team members engaged in ongoing, near-daily discussions through a dedicated group chat platform to address questions and ensure consistency in coding practices.

### Statistical Analysis

We evaluated the content of the encounters by measuring the mean, median, and IQR of words in each free-text encounter. We measured concordance between coders by calculating the proportion of encounters assigned the same *ICD-10* diagnosis code. We separately analyzed encounters in which both coders assigned a single diagnosis, both coders assigned multiple diagnoses, and at least 1 coder assigned multiple diagnoses. In cases of multiple diagnoses, we measured complete concordance (defined as all identical diagnoses), partial concordance (defined as at least 1 identical diagnosis), and complete discordance (defined as no identical diagnoses). We used chi-square tests of independence to compare concordance across single-diagnosis and multidiagnosis encounters. We determined the most common diagnoses and analyzed concordance stratified by patient characteristics using chi-square tests of independence for comparison across age, sex, and country of origin. We also used chi-square testing to compare concordance across encounters with different lengths of free-text documentation. We used R 2024.04.2 for all calculations.

## Results

From an original sample of 8546 encounters, we found that 99.0% (n=8460) were assigned at least 1 *ICD-10* code and therefore met inclusion criteria for analysis. These represented 5623 unique patients and 2774 unique diagnosis codes (Table 1). The diagnoses included communicable and noncommunicable illnesses (Table 2). The most common diagnoses included management of hypertension, diabetes mellitus, headaches, and encounters to receive prescription refills for chronic conditions. Common diagnoses that were likely related to infectious diseases during this study period included cough, fever, and urinary tract infection. The mean word count for free-text encounters was 20.5 words, with a median of 14 (IQR 2‐28) words per encounter. A sample of the free-text clinical documentation and diagnosis code assignments are shown in Table 3. There were 58.78% (4973/8460) encounters where both coders assigned 1 diagnosis code, 18.56% (1570/8460) encounters where both coders assigned multiple diagnosis codes, and 22.66% (1917/8460) encounters with a mixed number of codes assigned.

**Table 1.** Concordance of assigned *International Classification of Diseases, Tenth Revision* (*ICD-10*) diagnosis codes across demographic variables in free-text clinical encounters from humanitarian relief clinics in Mexico, 2021‐2022 (N=8460 encounters).

Characteristic	Proportion	Complete concordance	*P* value
Assigned diagnosis codes, n (%)			<.001
Single, both coders	4973 (58.78)	3096 (62.26)	
Multiple, both coders	1570 (18.56)	215 (13.69)	
Mixed, single and multiple	1917 (22.66)	N/A^a^	
Sex, n (%)			.15
Female	5203 (61.50)	1985 (38.15)	
Male	3257 (38.50)	1294 (39.73)	
Age group (years), n (%)			<.001
0‐5	827 (9.78)	350 (42.32)	
6‐10	776 (9.17)	333 (42.91)	
11‐20	538 (6.36)	210 (39.03)	
21‐30	1770 (20.92)	740 (41.81)	
31‐40	2827 (33.42)	1118 (39.55)	
>40	1722 (20.35)	528 (30.66)	
Country of origin, n (%)			.003
Brazil	235 (2.78)	101 (42.98)	
Chile	215 (2.54)	99 (46.05)	
Cuba	46 (0.54)	14 (30.43)	
El Salvador	846 (10.00)	343 (40.54)	
Guatemala	601 (7.10)	220 (36.61)	
Haiti	3157 (37.32)	1328 (42.07)	
Honduras	2126 (25.13)	760 (35.75)	
Mexico	247 (2.92)	98 (39.68)	
Unknown or other	987 (11.67)	316 (32.02)	
Free-text length quartiles, n (%)			<.001
0‐1 words (first quartile)	2095 (24.76)	1572 (75.04)	
2‐28 words (second and third quartile)	4179 (49.40)	1407 (33.67)	
29‐231 words (fourth quartile)	2186 (25.84)	300 (13.72)	

a N/A: not applicable.

**Table 2.** Most frequently assigned diagnoses in 8460 free-text clinical encounters from humanitarian relief clinics in Mexico, 2021‐2022^a^.

Diagnosis	*ICD-10*^b^ code	Frequency, n (%)
Primary hypertension	I10	1234 (14.59)
Type 2 diabetes mellitus	E11.9	639 (7.55)
Headache	R51.9	561 (6.63)
Repeat prescription	Z76.0	542 (6.41)
Cough	R05.9	500 (5.91)
Abdominal pain	R10.9	424 (5.01)
History of diseases of the circulatory system	Z86.79	346 (4.09)
Urinary tract infection	N39.0	345 (4.08)
Pain	R52	329 (3.89)
Fever	R50.9	305 (3.61)

a Incomplete records and those with nonspecific diagnosis codes (ie, Z00 codes) were excluded from this table.

b *ICD-10*: *International Classification of Diseases, Tenth Revision.*

**Table 3.** Sample free-text clinical encounters with assigned *International Classification of Diseases, Tenth Revision* (*ICD-10*) codes from humanitarian relief clinics in Mexico, 2021-2022.

Sex	Age group (years)	Country of origin	Free-text clinical documentation	Coder 1 diagnoses	Coder 2 diagnoses	Arbitrator diagnoses
Male	30-39	Mexico	“Sore throat × 7 days, green mucous, has been taking paracetamol × 7 days. Fever past 2 days. Last tylenol taken 11 pm. No allergies. Taking 2 other medications but can’t identify. Note HR. Double checked.”	J35.1, J02.9, R50.9	Z00.00	J35.1, J02.9, R50.9
Male	40-49	Honduras	“Pain and swelling in R eye and R neck and R side of head. Sun makes it worse. More than 1 year. Took ibuprofen.”	H10.31, R05.9	I10, H10.31	H10.31, I10
Female	60-69	Honduras	“Pt reports being seen here Tuesday for her foot pain and given pain medications at that time that have been working. Pt instructed to continue taking those meds PRN.”	R53.1, M79.673, R03.0	R53.1, M79.671	R26.89
Male	40-49	Nicaragua	“Cough productive of yellow phlegm and cold x4 days, afebrile 35.1 here, throat pain, no body aches, no vomiting or diarrhea, no sick contacts. large tonsils without exudates or erythema, lungs clear, nontoxic.”	J11.1, R05.9	J11.1, J35.1	R05.8, J00, R07.0
Female	40-49	Haiti	“Patient with diabetes and no insulin for 8 days. previously on metformin but didn’t tolerate secondary to nausea. now feels bad with blurred vision and headache. also has h/o ovarian cysts and some abdominal pain. PMH: DM, ovarian cysts.”	E11.9, N83.209, Z79.84	E08.620, E11.9	E11.9, N83.209, T38.3× 6A

Of the 4973 encounters with a single code, 62.26% (n=3096) were concordant and 37.74% (n=1877) were discordant. Of the 1877 discordant encounters, there were 68.67% (n=1289) of instances in which 1 coder was concordant with the arbitrator, and 31.33% (n=588) of instances that had a unique diagnosis assigned by the arbitrator which was not assigned by either coder. Therefore, only 11.82% (588/4973) single diagnosis encounters had a diagnosis that was not assigned by either of the initial 2 coders.

Of the 1570 encounters with multiple diagnosis codes, 13.69% (n=215) had complete concordance between the 2 coders, 64.65% (n=1015) were partially concordant, and 21.66% (n=340) were completely discordant. Of the 340 completely discordant encounters, there were 22.94% (n=78) of instances in which 1 coder was concordant with the arbitrator, 61.47% (n=209) of instances in which 1 coder was partially concordant with the arbitrator, and 15.59% (n=53) of instances that had unique diagnosis codes assigned by the arbitrator which were not assigned by either coder. Therefore, only 3.38% (53/1570) of multidiagnosis encounters had a diagnosis that was not assigned by either of the initial 2 coders.

The most frequent diagnoses in concordant clinical encounters were hypertension, acute upper respiratory tract infection, acute nasopharyngitis, viral infection, urinary tract infection, cough, and diabetes mellitus. The most frequent diagnoses in discordant clinical encounters were hypertension, headache, repeat prescription, diabetes mellitus, cough, abdominal pain, and history of diseases of the circulatory system. The frequency of concordance was not significantly different across sex categories: concordance was observed between 38.15% (1985/5203) of encounters with female patients and 39.73% (1294/3257) of encounters with male patients (*P*=.34). Concordance in diagnosis codes in encounters across age strata ranged from 30.66% to 42.91%. Clinical encounters with older patients (those older than 40 years) had lower concordance between coders than those with other age groups (*P*<.001). Concordance ranged from 30.43% to 46.05% in encounters across different countries of origin (Table 1).

Encounters with fewer words were more frequently coded in a concordant manner than encounters with more words (Table 1). Specifically, encounters with fewer than 2 words were coded concordantly 75.04% (1572/2095) of the time whereas encounters with greater than 28 words were coded concordantly 13.72% (300/2186) of the time. We found that *ICD-10* codes with less specificity (eg, Z00: Encounter for a General Examination Without Abnormal Findings) were more frequently assigned to encounters with 0 or 1 words.

## Discussion

In this analysis of clinical records from ad hoc humanitarian clinics serving displaced persons migrating in Mexico, we developed a system to assign medical diagnosis codes to clinical encounters. This system relied on entry-level medical coders to independently review encounters and then more senior medical coders to arbitrate differences between their assigned *ICD-10* codes. Our dataset included 8460 clinical encounters over a 16-month study period, representing 5623 unique patients. We found that the first 2 medical coders demonstrated concordance in more than 60% of their assignments of single- and multidiagnosis encounters. In cases where they differed, additional review by a third, more experienced coder allowed for resolution of most of the discordance. This novel method can be applied to other circumstances where it is necessary to review medical information collected through the course of medical relief operations.

Our analysis suggests that this approach is reliable across most patient demographic categories. As could be expected, encounters with multiple diagnoses were less frequently coded in a completely concordant manner than encounters with single diagnoses. Encounters with older patients in our sample had a lower proportion of their diagnosis codes completely concordant between assignments. This could be indicative of more comorbidities or more complex medical diagnoses in patients who are older. It is also possible that older adults had more frequent multidiagnosis encounters than younger patients, which could produce discrepancies in the diagnosis assigned based on the free-text medical encounter. While many of the same diagnoses were frequently assigned in both concordantly and discordantly coded encounters, some nonspecific diagnoses (eg, abdominal pain and history of disease of circulatory system) were more commonly assigned in discordantly coded encounters. There was also variation observed between the proportion of encounters with concordant diagnosis assignments across patients of different countries of origin; however, these differences were relatively small. It is possible that there are differences in complexity of the clinical documentation between people with different medical conditions and migration experiences. It is also possible that linguistic or communication barriers were present for some patients based on their country of origin, which may have impacted the clarity with which clinicians documented their evaluation.

We also noted a difference in how medical encounters were coded based on the length of free-text documentation in the medical record. Interestingly, clinical encounters with more text were less frequently coded in a concordant manner than encounters with fewer words. This may be because in encounters with few words, the clinicians frequently just stated the diagnosis rather than any of the clinical findings (eg, “pneumonia” or “hypertension”) whereas lengthy documentation in other encounters may describe several findings that could be coded in different ways. It is also likely that clinical encounters with lengthy documentation may systematically differ from less thoroughly documented encounters as the former may include greater complexity in their presentation, disease process, or management considerations.

To address these challenges, future adaptations of this approach should prioritize enhanced training for coders focused on complex cases, particularly those involving multidiagnosis encounters or nonspecific presentations common in low-resource settings. Future adaptations could also incorporate training on culturally specific presentations of diseases and variations in medical terminology across demographic groups [16]. This could help coders better understand and interpret free-text medical records originating from diverse populations. Collaborating with clinicians to improve the clarity and consistency of free-text documentation would further reduce ambiguities and support more accurate assignments, although this may not be feasible in many austere environments. Finally, periodic audits of coding concordance stratified by demographic characteristics could provide valuable feedback to ensure equity in coding across diverse patient populations.

There is growing use of electronic health records among relief organizations serving displaced persons worldwide [11,12,14]. Past reports from humanitarian settings have identified challenges in the implementation of *ICD-10* coding into existing health records, including the time required for data entry, staff turnover, and the operational capacity for data analysis [13,17,18]. The approach used in this study can be used to characterize medical diagnoses from electronic health record systems in disaster response and other austere settings with a high level of consistency [15]. The strengths of this approach include the reliability in coding and the ability to have records reviewed and coded remotely while relief operations are ongoing. It also has applicability to diverse geographic and sociopolitical contexts where clinicians work in low-resource environments. It should be noted that we have no way to ascertain diagnosis accuracy (ie, the “true” illnesses and conditions present in the clinical encounters studied). Our method of completing multiple reviews of each encounter presents challenges for efficiency with high-volume clinical operations. Future work may leverage large language models to more efficiently code clinical encounters, but this would require validation before implementation [12]. As with other operations involving electronic health records, patient privacy and data security are also paramount concerns [16].

There are limitations in this analysis and approach. First, given that this analysis was conducted retrospectively, we cannot compare the accuracy of diagnostic codes with the reference standard of clinician designation, nor is this information readily available from clinics in austere environments. In addition, because of the limited diagnostic capabilities in the clinical sites studied, most of the diagnoses were based on the bedside examination (ie, history and examination), which resulted in nonspecific diagnoses being assigned.

In summary, we developed a system to assign *ICD-10* codes to thousands of clinical encounters for displaced people in transit at the Mexico-US border, with a variety of clinical presentations for a diverse patient population. The simple methodological approach described in this report holds potential to be adapted to other disaster response and resource-limited settings to advance the provision of clinical care and population health research. Scale-up of the electronic health record technology described in this study can improve patient safety by allowing program managers to monitor quality of care of diagnoses across patient populations. It can also provide critical information to public health officials regarding risk and clusters of transmissible infections, trauma and injuries, and outbreak preparedness.

## Abbreviations

*CPT*: *Current Procedural Terminology*
fEMR: Fast Electronic Medical Record
*ICD-10*: *International Classification of Diseases, Tenth Revision*
